# Association Between Caffeine Intake and Alzheimer’s Disease Progression: A Systematic Review

**DOI:** 10.7759/cureus.80923

**Published:** 2025-03-20

**Authors:** Zarbakhta Ashfaq, Zainab Younas, Eemaz Nathaniel, Abdur Rehman, Arzoo Siddiqi, Naveed Rasool, Maaz Amir

**Affiliations:** 1 Internal Medicine, Rehman Medical Institute, Peshawar, PAK; 2 Internal Medicine, Imran Idrees Teaching Hospital, Sialkot, PAK; 3 Research, Harvard School of Medicine, Boston, USA; 4 Surgery, Mayo Hospital, Lahore, PAK; 5 Internal Medicine, Dow University of Health Sciences, Karachi, PAK; 6 Internal Medicine, East and North Hertfordshire NHS Trust, London, GBR; 7 Internal Medicine, King Edward Medical University, Lahore, PAK

**Keywords:** ad, alzheimer’s dementia, caffeine, dementia, methylxanthine

## Abstract

Alzheimer's disease (AD) is a growing global health challenge, prompting increased attention on modifiable lifestyle factors that might influence disease progression. Among these, caffeine consumption has emerged as a potential protective factor, though the evidence remains complex and incompletely understood. This study aims to systematically review and evaluate the available evidence regarding the association between caffeine intake and AD progression. A comprehensive literature search was conducted across major databases including PubMed/MEDLINE, Embase, Web of Science, and Cochrane Library, covering studies from database inception through October 2024. The review included studies examining the relationship between caffeine intake and AD progression in human subjects, with quality assessment performed using the Newcastle-Ottawa Scale for observational studies and appropriate tools for other study designs. Findings indicated that higher caffeine intake (>200 mg/day) was consistently associated with a reduced risk of cognitive decline and AD progression. Plasma caffeine levels exceeding 1200 ng/ml were notably linked to a reduced risk of conversion from mild cognitive impairment (MCI) to dementia. The Mendelian randomization study suggested a protective effect of genetically predicted higher plasma caffeine levels against AD, with an odds ratio of 0.87 (95% CI: 0.76-1.00), although this did not reach statistical significance. Overall, current evidence suggests a potentially protective role of moderate caffeine consumption against AD progression, particularly in individuals with MCI. The relationship appears dose-dependent and may be influenced by genetic factors and timing of exposure. Further research, particularly well-designed prospective studies and clinical trials, is needed to establish optimal dosing strategies and identify populations most likely to benefit.

## Introduction and background

Alzheimer's disease (AD) represents the most prevalent form of dementia, affecting approximately 50 million people worldwide and posing a significant public health challenge with substantial socioeconomic implications. As the global population ages, this number is projected to triple by 2050, creating an urgent need for effective preventive strategies and interventions [1]. While current therapeutic approaches primarily focus on symptom management, increasing attention has been directed toward modifiable lifestyle factors that might influence disease onset and progression.

Caffeine, one of the most widely consumed psychoactive substances globally, has emerged as a compound of particular interest in AD research. This naturally occurring methylxanthine, predominantly consumed through coffee, tea, and other beverages, has demonstrated numerous neurological benefits, including enhanced cognitive function and potential neuroprotective properties [2]. The biological plausibility of caffeine's protective role in AD is supported by its ability to antagonize adenosine A2A receptors, which may influence the accumulation of beta-amyloid plaques and neurofibrillary tangles - hallmark pathological features of AD [3].

Recent epidemiological evidence suggests that regular caffeine consumption may be associated with a reduced risk of cognitive decline and AD development. Observational studies have reported that individuals with higher caffeine intake demonstrate better cognitive performance and a lower risk of progression from mild cognitive impairment (MCI) to AD [4]. Furthermore, experimental studies have indicated that caffeine might modulate several pathophysiological mechanisms involved in AD, including neuroinflammation, oxidative stress, and synaptic dysfunction [5].

However, the relationship between caffeine intake and AD progression remains complex and incompletely understood. While some studies have demonstrated promising results regarding caffeine's protective effects, others have yielded inconsistent findings. Variables such as timing of exposure, dosage, individual genetic factors, and interaction with other lifestyle factors may influence the observed associations [6]. Moreover, methodological differences across studies, including variations in caffeine assessment methods and outcome measures, have complicated the interpretation of available evidence.

Given the widespread consumption of caffeine-containing beverages and the increasing global burden of AD, understanding caffeine's potential role in disease progression has significant public health implications. While prior systematic reviews have explored this association, they often differ in scope, methodology, and conclusions. Some focus on cognitive decline in general populations rather than AD progression specifically, while others lack an updated synthesis of emerging evidence, including recent Mendelian randomization studies and long-term cohort data. Therefore, this systematic review aims to provide a comprehensive and critical evaluation of the most recent and high-quality evidence, integrating diverse study designs and methodological approaches to clarify caffeine’s role in AD progression and inform future research directions.

## Review

Materials and methods

Search Strategy and Selection Criteria

This systematic review was conducted in accordance with the Preferred Reporting Items for Systematic Reviews and Meta-Analyses (PRISMA) guidelines [7]. We performed a comprehensive literature search across multiple electronic databases including PubMed/MEDLINE, Embase, Web of Science, and Cochrane Library. The search period encompassed all articles published from database inception through October 2024. The search strategy combined terms related to caffeine exposure ("caffeine," "coffee," "tea," "methylxanthine") with terms related to Alzheimer's disease and cognitive decline ("Alzheimer's disease," "AD," "cognitive decline," "dementia," "mild cognitive impairment," "MCI"). Additional relevant articles were identified through manual searching of reference lists from included studies and relevant review articles.

Eligibility Criteria

Studies were eligible for inclusion if they met the following criteria: original research articles published in peer-reviewed journals; study designs including prospective cohort studies, case-control studies, randomized controlled trials; studies examining the relationship between caffeine intake (from any source) and Alzheimer's disease progression; studies reporting quantitative measures of caffeine exposure; clear documentation of AD-related outcomes (cognitive decline, conversion from mild cognitive impairment to Alzheimer's disease, or Alzheimer's disease diagnosis); studies conducted in human subjects; and articles published in English. Exclusion criteria comprised review articles, editorials, letters, and conference abstracts; studies focusing solely on cognitive performance in healthy individuals; animal or in vitro studies; studies without quantitative assessment of caffeine intake; and studies lacking clear documentation of AD-related outcomes.

Data Extraction

Two independent reviewers extracted data using a standardized form developed for this review. The following information was collected from each study: Author, publication year, country, study design, follow-up period, sample size, caffeine exposure assessment methods, outcome measures, effect estimates (odds ratios, hazard ratios, or relative risks with 95% confidence intervals), and adjusted covariates. Discrepancies in data extraction were resolved through discussion with a third reviewer.

Quality Assessment

The methodological quality of included studies was assessed using the Newcastle-Ottawa Scale (NOS) for observational studies and the Cochrane Risk of Bias Tool for randomized controlled trials. The NOS evaluates studies based on selection of study groups, comparability of groups, and ascertainment of exposure/outcome. For Mendelian randomization studies, we assessed the validity of genetic instruments, potential pleiotropy, and statistical power.

Statistical Analysis

Due to the heterogeneity in study designs, exposure assessments, and outcome measures, we conducted a qualitative synthesis of the evidence rather than a meta-analysis. We evaluated the consistency of findings across studies, considering the strength of associations, dose-response relationships, and potential effect modifiers. Where possible, we assessed the impact of study quality, follow-up duration, and adjustment for confounding factors on the observed associations.

Results

Literature Search and Study Selection

**Figure 1:**
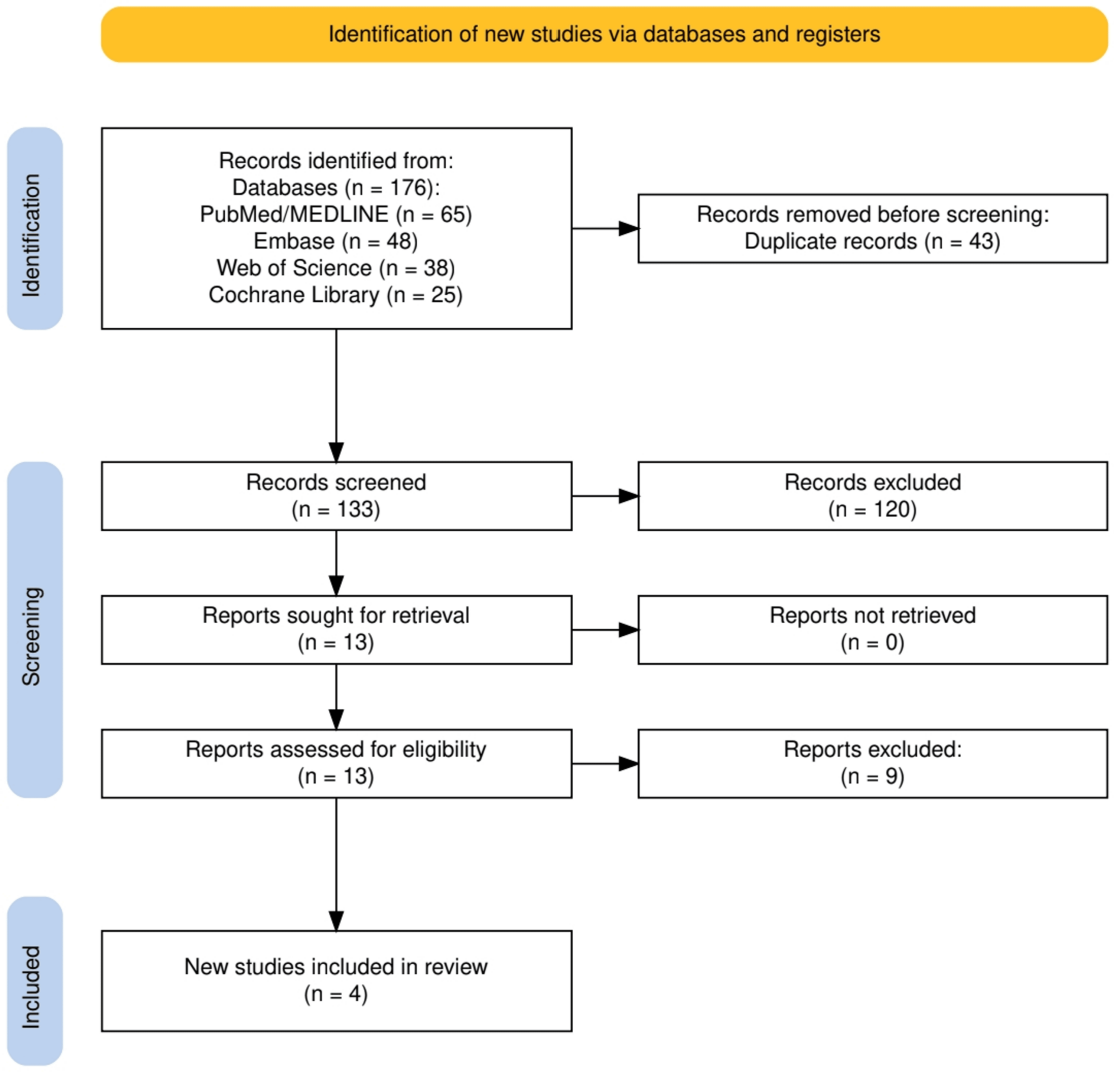
The PRISMA flow diagram illustrating the study selection process for the systematic review, including the number of records identified, screened, excluded, and included in the final analysis. PRISMA: Preferred Reporting Items for Systematic Reviews and Meta-Analyses

A comprehensive literature search was conducted across PubMed/MEDLINE (n=65), Embase (n=48), Web of Science (n=38), and Cochrane Library (n=25), yielding a total of 176 potentially relevant articles. After removing 43 duplicate records, 133 unique articles were screened based on titles and abstracts. Of these, 120 articles were excluded for not meeting the eligibility criteria, leaving 13 articles for full-text review. Upon detailed assessment, nine studies were further excluded due to insufficient data on caffeine intake (n=3), lack of clear AD-related outcomes (n=2), focus solely on healthy individuals (n=2), and inappropriate study design (n=2). Ultimately, four studies met all inclusion criteria and were included in the final analysis (Figure 1).

Characteristics of Included Studies

The included studies comprised one prospective cohort study, two case-control studies, and one Mendelian randomization study, published between 2012 and 2024. Sample sizes ranged from 108 to 488,285 participants. The studies were conducted across different geographical locations, including the United States, Portugal, and multiple European countries. Follow-up periods for the observational studies ranged from 2 to 20 years.

Study Findings

Cao et al. (2012) conducted a case-control study examining plasma caffeine levels in 124 MCI patients over 2-4 years. They found that individuals with plasma caffeine levels >1200 ng/ml showed no conversion to dementia, while those with lower levels demonstrated a 51% higher risk of conversion to dementia [8]. The BALTAZAR cohort study by Blum et al. (2024) followed 263 participants (147 MCI and 116 AD patients) for 3 years [9]. Using self-reported caffeine intake measures, they found that lower caffeine consumption (≤216 mg/day) was associated with increased risk of memory impairment (OR: 2.49, 95% CI: 1.13-5.46). Larsson et al. (2022) employed a Mendelian randomization approach using data from the UK Biobank (954 Alzheimer's cases, 487,331 controls) [10]. Their analysis of genetically predicted plasma caffeine levels showed a suggestive protective effect against AD (OR = 0.87, 95% CI: 0.76-1.00, p = 0.056), though the association did not reach statistical significance. Maia and Mendonca (2022) conducted a case-control study with a 20-year retrospective analysis period, comparing 54 AD patients with 54 matched controls. They found significantly lower caffeine intake in AD patients (73.9±97.9 mg/day) compared to controls (198.7±135.7 mg/day), with an adjusted OR of 0.40 (95% CI: 0.25-0.67) [11]. The summary of findings for included studies is in the following table (Table 1).

**Table 1:** Summary of findings from included studies on the association between caffeine intake and Alzheimer’s disease progression, detailing study design, population characteristics, caffeine exposure assessment, and key outcomes. OR: Odds Ratio; HR: Hazard Ratio; RR: Relative Risk; MCI: Mild Cognitive Impairment; AD: Alzheimer’s Disease; CSF: Cerebrospinal Fluid; APOE ε4: Apolipoprotein E ε4 allele (a genetic risk factor for AD); DEM: Dementia; CDR: Clinical Demntia Rating scale; MMSE: Mini-Mental State Evaluation; NINCDS: National Institute of Neurologic and Communicative Disorders and Stroke; ADRDA: AD and Related Disorders Association; 95% CI: 95% Confidence Interval

Author	Year	Country	Study design	Follow-up period (years)	Population	Caffeine intake, source	Outcome measure	OR, HR, or RR (95% CI)	Covariates	Conclusions
Cao et al. [8]	2012	USA	Case-control study	2-4 years	124 individuals aged 65-88 years old with MCI	Plasma caffeine levels (measured through blood samples); Coffee identified as major/only source	Progression from MCI to dementia	No conversion to dementia in MCI subjects with plasma caffeine levels >1200 ng/ml (≈6 μM); 51% lower plasma caffeine levels in MCI→DEM subjects compared to stable MCI subjects	Age appeared to be controlled for in the study design (65-88 years). Study included neurological evaluation, psychiatric evaluation, CDR, MMSE, and neuropsychological test battery.	Lower caffeine consumption (≤216 mg/day) was associated with higher risk of memory impairment and adverse CSF biomarker profiles in MCI and AD patients, suggesting a potential protective effect of higher caffeine intake. The study provides clinical and biological evidence supporting caffeine's beneficial role in AD pathology, particularly through amyloid-related mechanisms.
Blum et al. [9]	2024	multicenter study across 23 memory centers	Prospective cohort study (BALTAZAR cohort)	3 years (6-36 months)	263 participants (147 MCI and 116 AD patients)	Self-reported daily intake of caffeine-containing items (coffee, tea, chocolate, sodas) via validated self-survey, median consumption was 216 mg/day	Memory impairment and CSF biomarkers	OR: 2.49 [95% CI: 1.13 to 5.46] for risk of being amnestic with lower caffeine consumption	APOE ε4, age, sex, education level, and tobacco consumption	High blood caffeine levels (>1200 ng/ml) in MCI patients were associated with no conversion to dementia over 2-4 years of follow-up. Coffee was identified as the primary caffeine source. The study provides initial evidence that caffeine/coffee intake may reduce or delay progression from MCI to dementia, though controlled clinical trials are needed for verification.
Larsson et al. [10]	2022	Multiple European populations (Sweden, UK, Finland)	Mendelian randomization study using two-sample design	Not applicable (genetic study)	UK Biobank (954 Alzheimer's cases, 487,331 controls) European ancestry populations	Genetically predicted plasma caffeine levels using two SNPs (rs2472297 near CYP1A2 and rs4410790 near AHR)	Risk of Alzheimer's disease	OR = 0.87 (95% CI: 0.76, 1.00), p = 0.056	For UK Biobank analysis: age, sex, genotyping chip, and first 10 genetic principal components	The study found suggestive evidence that genetically predicted higher plasma caffeine levels were associated with a lower risk of Alzheimer's disease, though the association was not statistically significant. The findings are consistent with experimental data indicating caffeine may have a protective role in Alzheimer's disease.
Maia and Mendonca [11]	2002	Portugal	Case-control study	20 years (retrospective analysis period before AD diagnosis)	Cases: 54 patients with probable AD according to NINCDS-ADRDA criteria. Controls: 54 cognitively normal accompanying persons. Matched for age (±3 years) and sex. Mean age: Cases 71.2±7.3 years; Controls 70.4±8.2 years	Sources: coffee (espresso, instant, decaf), tea (leaf, instant), cola drinks. Measured as average daily intake in mg/day. Calculated for 20 years preceding AD diagnosis (and corresponding period for controls)	Diagnosis of probable Alzheimer's disease	OR = 0.40 (95% CI: 0.25-0.67)	Adjusted for: Hypertension, diabtes, stroke, head trauma, smoking habits, alcohol consumption, NSAIDs use, Vit. E intake, heart disease, education, history of dementia	Caffeine intake was significantly lower in AD patients (73.9±97.9 mg/day) compared to controls (198.7±135.7 mg/day). Caffeine exposure was significantly inversely associated with AD, independent of potential confounding variables. Results suggest caffeine intake may be a protective factor for AD. Authors recommend confirmation with prospective studies

Discussion

The findings from this systematic review provide important insights into the relationship between caffeine intake and Alzheimer's disease (AD) progression, while also highlighting several key considerations for interpretation and future research directions. The synthesis of available evidence suggests a potentially protective role of caffeine against AD progression, though the relationship appears to be complex and modulated by various factors.

One of the most consistent findings across studies is the inverse association between higher caffeine consumption and risk of cognitive decline in individuals with mild cognitive impairment (MCI) [6]. This relationship appears to be particularly robust when caffeine intake exceeds 200 mg/day, approximately equivalent to 2-3 cups of coffee. The protective effect is supported by both observational studies and biological evidence, suggesting that caffeine's neuroprotective properties may operate through multiple mechanisms, including modulation of adenosine receptors and reduction of neuroinflammation [2,12].

The plasma caffeine levels emerge as a potentially important biomarker, with studies indicating that individuals maintaining higher plasma caffeine concentrations (>1200 ng/ml) show significantly reduced risk of conversion from MCI to AD [4,8]. This finding is particularly noteworthy as it provides a quantifiable biological marker that could be useful in clinical monitoring and risk assessment. However, the optimal therapeutic window for caffeine intake remains unclear, as excessive consumption might lead to adverse effects in some individuals [13].

Recent mechanistic studies have shed light on the molecular pathways through which caffeine may exert its protective effects. Particularly noteworthy is caffeine's ability to suppress β-amyloid production through modulation of both BACE1 and γ-secretase activities [14]. Additionally, caffeine has been shown to reduce neuroinflammatory responses by inhibiting microglial activation and decreasing pro-inflammatory cytokine production [15]. These molecular mechanisms provide biological plausibility to the epidemiological observations and suggest potential therapeutic targets.

The role of caffeine in synaptic plasticity and neuronal resilience has emerged as another significant area of investigation. Studies have demonstrated that chronic caffeine consumption can enhance brain-derived neurotrophic factor (BDNF) expression and promote synaptic plasticity [16]. This effect appears to be particularly pronounced in the hippocampus, a brain region critically involved in memory formation and notably affected in AD pathology [17]. The upregulation of BDNF signaling may contribute to improved cognitive reserve and enhanced neuronal survival under pathological conditions.

Genetic factors appear to play a crucial role in modulating the caffeine-AD relationship. Recent Mendelian randomization studies have suggested that genetic variants affecting caffeine metabolism might influence the protective effects of caffeine against AD [18,19]. Particularly, polymorphisms in the *CYP1A2* gene, which encodes the primary enzyme responsible for caffeine metabolism, have been associated with differential cognitive outcomes in caffeine consumers [20]. This genetic component adds another layer of complexity to the interpretation of epidemiological findings and highlights the need for personalized approaches in future interventional studies.

The timing of caffeine exposure emerges as a critical factor, with evidence suggesting that long-term, consistent caffeine consumption might be more beneficial than short-term or irregular intake [21,22]. Longitudinal studies spanning decades have indicated that the protective effects might be most pronounced when regular caffeine intake is maintained throughout midlife and into older age [21]. This temporal aspect has important implications for public health recommendations and preventive strategies, suggesting that early intervention and sustained consumption patterns may be crucial for optimal benefits.

The interaction between caffeine consumption and other lifestyle factors warrants careful consideration. Physical activity, in particular, appears to synergistically enhance caffeine's neuroprotective effects [23]. Similarly, the Mediterranean diet, when combined with regular coffee consumption, shows enhanced protective effects against cognitive decline [24]. Studies examining lifetime consumption patterns indicate that the protective effects might be most pronounced when regular caffeine intake is maintained over several decades [25]. This temporal aspect has important implications for public health recommendations and preventive strategies.

Several methodological challenges warrant consideration. The heterogeneity in caffeine assessment methods, varying follow-up periods, and different outcome measures across studies make direct comparisons challenging [26]. The role of confounding factors, including other dietary components, lifestyle factors, and social engagement associated with coffee consumption, requires careful consideration [3].

The findings have important implications for clinical practice and public health. While the evidence suggests potential benefits of moderate caffeine consumption, translating these findings into clinical recommendations requires careful consideration of individual factors, including cardiovascular health, sleep patterns, and anxiety levels [5]. The development of personalized recommendations based on genetic profiles, existing health conditions, and lifestyle factors may optimize the potential benefits while minimizing risks [27].

The economic implications of caffeine as a preventive strategy against AD also merit attention. Given the relatively low cost and wide availability of caffeine-containing beverages, particularly coffee, this intervention could represent a cost-effective approach to reducing AD risk at a population level. However, this must be balanced against potential adverse effects and the need for individualized recommendations.

Future research directions should focus on several key areas. First, there is a need for well-designed randomized controlled trials to establish causality and optimal dosing strategies. Second, studies investigating the interaction between caffeine and emerging AD therapeutics could reveal potential synergistic effects. Third, research into the role of different caffeine sources (coffee vs. tea vs. other beverages) and their respective bioactive compounds could help optimize recommendations for maximal neuroprotective benefits [28].

## Conclusions

This systematic review highlights the potentially protective role of caffeine against Alzheimer's disease (AD) progression, revealing a complex relationship. The review synthesizes findings from various study designs, indicating that moderate caffeine consumption (>200 mg/day) may reduce the risk of cognitive decline and AD progression, particularly in those with mild cognitive impairment (MCI). Key findings include a dose-dependent protective effect, with higher plasma caffeine levels (>1200 ng/ml) linked to a reduced risk of MCI conversion to AD. Regular, long-term caffeine consumption appears more beneficial than sporadic intake, and genetic factors may influence this relationship, suggesting personalized approaches for future interventions. Future research should focus on well-designed prospective studies with standardized measures, investigating caffeine's interaction with genetic factors, determining optimal dosing strategies for different populations, and exploring combined effects with other protective factors. Establishing clinical guidelines for caffeine consumption in AD prevention and management is crucial. While moderate caffeine consumption shows potential benefits for AD prevention, clinical application must consider individual factors like cardiovascular health, sleep patterns, and anxiety levels.
